# Tuberculosis in South Asia: a tide in the affairs of men

**DOI:** 10.1186/s40248-018-0122-y

**Published:** 2018-03-22

**Authors:** Buddha Basnyat, Maxine Caws, Zarir Udwadia

**Affiliations:** 1 Centre for Tropical Medicine and Global Health, University of Oxford, Oxford, UK; 2 Oxford University Clinical Research Unit, Patan Academy of Health Sciences, Lalitpur, Nepal; 3 Global Antibiotic Resistance Partnership, Kathmandu, Nepal; 4 Liverpool School of Tropical Medicine, Liverpool, UK; 5 British Nepal Medical Trust, Kathmandu, Nepal; 6 Hinduja Hospital and Research Center, Mumbai, India

**Keywords:** Infectious disease, LMICs, Equity, Tuberculosis, Research priorities

## Abstract

**Background:**

Tuberculosis (TB) remains the most common cause of infectious disease deaths worldwide. What is perhaps less appreciated is that the caseload of tuberculosis patients in South Asia is staggering.

South Asia has almost 40% of the global TB burden with 4,028,165 cases in 2015. This region also has a disproportionate share of TB deaths (681,975 deaths, 38% of the global burden). Worldwide just 12.5% of TB cases are in HIV positive individuals, but much research and investment has focused on HIV-associated TB. Only 3.5% of patients with tuberculosis in South Asia have HIV co-infection. Not surprisingly with such a huge burden of disease, this region has an estimated 184,336 multi drug resistant (MDR) cases among notified TB cases which accounts for a third of global MDR burden. Crucially, at least 70% of the estimated MDR cases remain untreated in this region and MDR treatment success ranged from only 46% for India to 88% for Sri Lanka in the 2012 cohort that received treatment. This region represents many of the drivers of the modern TB epidemic: rapid urbanization and high density populations with dramatically rising incidence of diabetes, a burgeoning and largely unregulated private sector with escalating drug resistance and high air pollution both outdoor and household.

**Conclusion:**

From bacterial biochemistry to policy implementation, we suggest ways in which South Asia can seize the opportunity lead global TB elimination by demonstrating feasibility in some of the world’s most densely populated cities and remotest reaches of the Himalayas. Clearly political will is essential, but we cannot defeat TB without understanding how to eliminate it in South Asia.

## Background

Although tuberculosis (TB) remains the most common cause of infectious disease deaths worldwide, impressive gains have been made in TB control since the millennium. Globally TB mortality fell by 47% between 1990 and 2015 [1]. Sixteen out of the 22 high TB burden countries achieved the millennium development goals for TB. The global health community has set a laudably ambitious target for the post-2015 END-TB strategy of a 25% reduction in incidence and a 75% reduction in mortality between 2015 and 2025, and by 2035 a 95% reduction in mortality and 90% reduction in incidence [1]. In 1967, when the decision was taken to push for smallpox eradication, most believed it could not be done. There were at that time 3 million deaths each year from smallpox- a higher mortality than that of TB today. Although TB elimination is a much more complex problem, our understanding of infectious diseases, global connectivity and range of interventions has also advanced. To accelerate TB elimination, we must maximize research in high burden regions, and learn from failures to improve translation, innovation and adoption to scale-up. South Asia has the potential to act as a model region for TB research and the drive towards elimination.

**Fig. 1:**
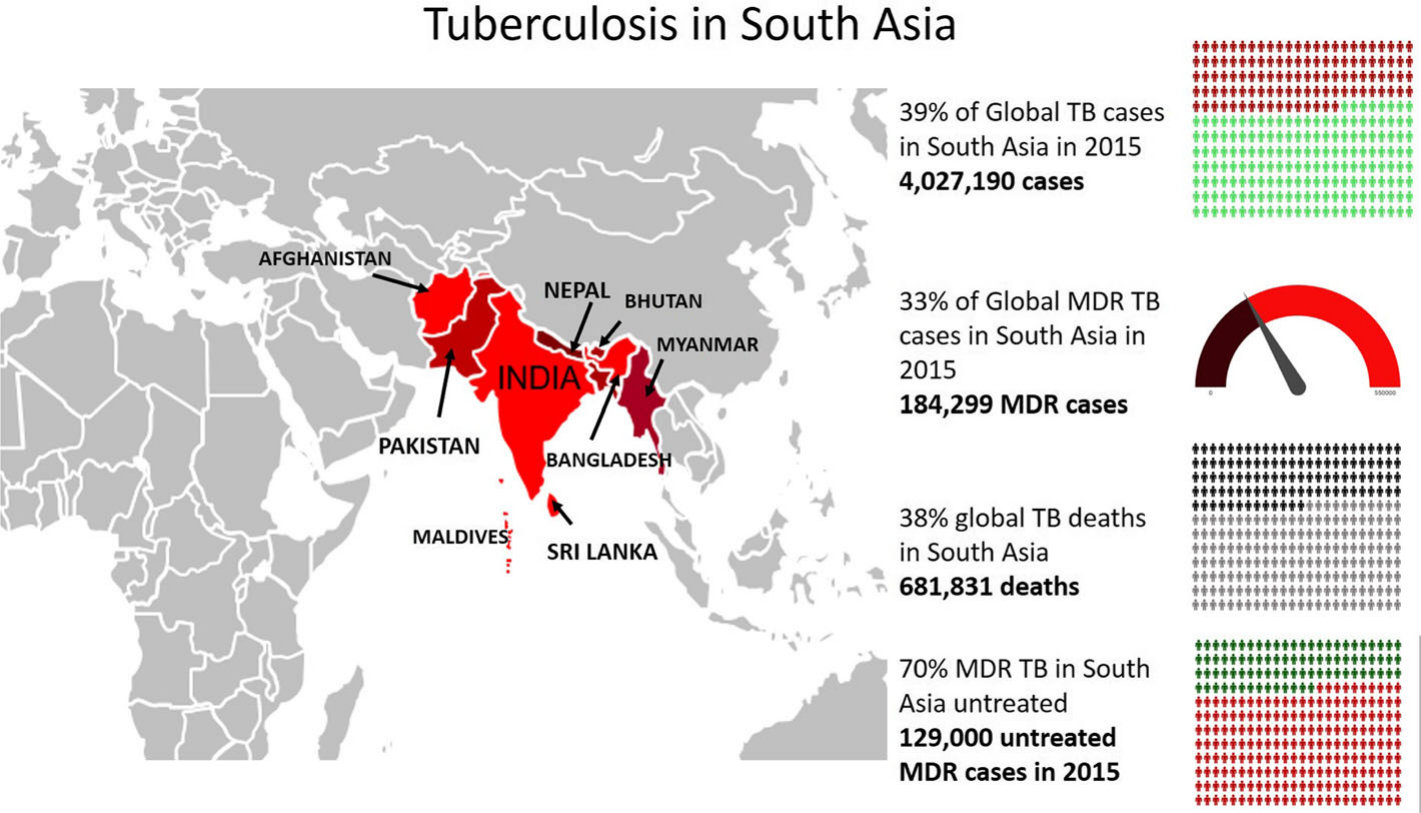
Tuberculosis in South Asia

The caseload (Table 1, Fig. 1) of tuberculosis patients in South Asia (India, Pakistan, Bangladesh, Bhutan, Sri Lanka, Nepal, Afghanistan, Myanmar, and Maldives) is staggering and not well appreciated [2]. South Asia has almost 40% of the global TB burden with 4,028,165 cases in 2015 [2]. This region also has a disproportionate share of TB deaths (681,975 deaths, 38% of the global burden). Worldwide just 12.5% of TB cases are in HIV positive individuals, but much research and investment have focused on HIV-associated TB. Only 3.5% of patients with tuberculosis in South Asia have HIV co-infection (141,839/4,027190) [2]. This reflects the low prevalence of HIV in the general population in South Asia, where the epidemic is focused in high risk groups such as intravenous drug users, commercial sex workers, men who have sex with men and long distance truck drivers. Population prevelance for young adults (15–49 years) in the general population is below 1% for all countries, and below 0.1% for four (Table 1). Not surprisingly with such a huge burden of TB disease, this region has an estimated 184,336 multi drug resistant (MDR) cases among notified TB cases which accounts for a third of global MDR burden [2]. Crucially, at least 70% of the estimated MDR cases remain untreated in this region and MDR treatment success ranged from only 46% for India to 88% for Sri Lanka in the 2012 cohort. This region represents many of the drivers of the modern TB epidemic: rapid urbanization and high density populations with dramatically rising incidence of diabetes [3, 4], a burgeoning and largely unregulated private sector and escalating drug resistance. Factors affecting lung health and therefore increasing susceptibility to TB are also of increasing significance in South Asia: surging tobacco use [3], occupational lung disease and high air pollution both outdoor and household due to wood and dung as cooking fuel [5, 6]. We cannot defeat TB without understanding how to eliminate it in South Asia.

**Table 1:** Tuberculosis in South Asia in 2015

Country	TB Incidence (per 100,000 population)	Absolute number of new TB cases 2015 (N %)	HIV prevalence general population^a^	Absolute number of TB cases in HIV infected individuals 2015	Absolute number of MDR TB cases 2015	Absolute number of deaths from TB 2015
Afghanistan	189	61,000 (1.5)	< 0.1	460 (0.3)	3000 (1.63)	12,170 (1.8)
Bangladesh	225	362,000 (9.0)	< 0.1	630 (0.4)	9700 (5.3)	73,230 (10.7)
Bhutan	155	975 (0.02)	No estimate	6 (0.004)	37 (2.0)	144 (0.02)
India	217	2,840,000 (70.5)	0.3	113,000 (79.7)	130,000 (70.5)	517,000 (75.8)
Nepal	156	44,000 (1.1)	0.2	1900 (1.3)	1500 (0.8)	1900 (0.3)
Maldives	53	190 (0.004)	No estimate	0 (0)	10 (0.005)	20 (0.003)
Myanmar	365	197,000 (4.9)	0.8	17,000 (12.0)	14,000 (7.6)	27,000 (4.0)
Pakistan	270	510,000 (12.7)	0.1	8800 (6.2)	26,000 (14.1)	45,600 (6.7)
Sri Lanka	65	13,000 (0.3)	< 0.1	43 (0.03)	89 (0.05)	1211 (0.2)
South Asia Total		4,028,165 (100)		141,839 (100)	184,336 (100)	681,975 (100)

^a^Adults aged 15–49. Source UNAIDS data. Available at: http://aidsinfo.unaids.org/

Modelling studies suggest that even with full scale-up of existing interventions using unlimited resources, the END TB targets are not achievable in India; the challenges in other countries in the region are even greater [7]. There is often a substantial gap between policy and practical implementation [8]. These studies underline the need to improve our fundamental armoury in the fight for TB elimination through strengthened and sustained research capacity in high burden countries.

South Asia was a key player in the early clinical trials optimizing TB case management, including the famous ‘Madras’ study [9]. The last decade has again seen many notable contributions to TB control from South Asia, there is a clear opportunity to build on these achievements. Only by investing in, scaling up and strategically networking our research efforts in South Asia we can optimize effective multifaceted interventions to achieve TB elimination in Asia.

The zero TB cities initiative in Chennai represents one example of innovative multifaceted research in the region. By investing in strategically planned research and implementation now, we can seize the tide and advance the tools we need to achieve the ambitious targets we have set ourselves. From active case finding, diagnosis and treatment to bacterial biochemistry to research priorities and policy implementation, in this presentation, we suggest ways in which South Asia can seize the opportunity to help in leading global TB elimination by demonstrating feasibility in some of the world’s most densely populated cities and remotest reaches of the Himalayas.

## Diagnosis and active case finding

The 2016 WHO global TB report has substantially revised estimates of TB burden in India upwards [2]. Evidence was collated from a number of sources, including a statewide prevalence survey in the relatively wealthy state of Gujarat which showed prevalence of TB 50% higher than previously estimated. This means in turn that case notification is substantially lower than previously estimated, revised downwards to 59% rather than 74% [2]. It is likely that prevalence surveys in other countries of the region, including the one just launched in Nepal, will find similar underestimates of burden. The keystone of epidemic control for infectious diseases, from smallpox to SARS and ebola, has always been case identification and interruption of transmission. While the roll-out of the molecular GeneXpert test has improved TB diagnosis and it is hoped it will reduce the diagnostic gap, population coverage remains low in South Asia [10]. Table 2 summarises the availability of GeneXpert in 2016 South Asian countries. As of 31^st^ December 2016, the region averaged 0.28 GeneXpert modules procured per 100,000 population. The availability GeneXpert modules are generally concentrated in large urban centres and average data masks regional variability and further overestimates coverage due to issues with maintenance of installed modules. Despite the huge burden of tuberculosis in this region and national policies which often include contact tracing, in routine practice there has been an almost exclusive focus on passive case finding, with diagnosis and treatment of only the index patient [11]. Due to resource constraints, there has been until recently very little emphasis on active case finding (screening individuals at increased risk of the disease) in susceptible populations such as household contacts of patients with tuberculosis or in areas of highest transmission (for example cities in South Asia [12, 13]) so that treatment can be begun and the patient rendered non- infectious [14]. While this is now widely recognized as a major gap in TB control activities, short term ‘demonstration’ projects or pockets of intensified activity must translate to sustained comprehensive national active case finding integration to national TB programmes [11]. Innovative public-private engagement in Pakistan funded initially by TB REACH has recently been awarded over 40 million USD from the Global Fund to scale up nationwide and integrate it within national TB control activities [15]. The next step is to adapt such successful projects to other countries in the region for evaluation and scale-up.

**Table 2:** GeneXpert availability in countries of South Asia as of 31^st^ December 2016(http://apps.who.int/tb/laboratory/xpertmap/)

Country	No of Xpert modules procured	Total population (thousands, 2016)^a^	Modules per 100, 000 population	No. of Xpert cartridges procured
Afghanistan	42	34,656.03	0.12	10,770
Bangladesh	424	162,951.56	0.26	381,210
Bhutan	12	797.76	1.23	2600
India	3000	1,324,171.35	0.23	2,134,760
Maldives	4	417.49	0.96	500
Myanmar	416	52,885.22	0.79	340,370
Nepal	143	28,982.77	0.49	136,170
Pakistan	1012	193,203.48	0.52	525,500
Sri Lanka	62	21,203.00	0.29	13,100
Total	5115	1,819,268.66	0.28	n/a

^a^Source: UN population division: https://esa.un.org/unpd/wpp/

South Asia has a massive, confusing and unregulated private health sector. The vast majority of hospitals in the region are privately run with a great mix of alternative disciplines like homeopathy, Tibetan and Ayurveda. Pakistan, for example, has a very rich tradition in the use of medicinal plants for the treatment of various ailments based predominantly on the *Unani* system of medicine, which dates back to the Indus valley civilization [16]. Private practitioners often begin the initial management of tuberculosis, with increasing evidence that the role of the private sector is larger than previously suspected.

Mandatory case notification and national web-based reporting introduced in India in 2012 generated a 34% in annual case notifications in 2015 compared to 2013. This system should be replicated regionally. India, Bangladesh and Pakistan are three out of the ten countries globally with the biggest gap between TB notification and estimated incidence.

In addition to missed opportunities for case notification, a largely unregulated private sector has led to widespread misdiagnosis and treatment of TB cases. The consequences for individual patients are severe, both in terms of increased morbidity and financial burden. From a wider public health perspective, this leads to generation and propagation of drug resistant strains of TB. The first report of totally drug resistant TB came from India in 2012 [17]. Although this term is not recognized by the World Health Organization due to difficulties of definition [18], the report reflects South Asia as an incubator for drug resistant forms of TB with an ever increasing spectrum of resistance.

Poor access to TB diagnostic services, which can be caused by many factors including financial barriers, transport, clinic opening hours and stigma, led to the widespread use of inaccurate commercial serological diagnostic tests for TB. Despite their lack of accuracy, these tests offer simple, rapid and economical ‘TB testing’ at pharmacies or cornershops. In many cases equivalent as a diagnostic aid to tossing a coin, use of these tests results in both delayed diagnosis for TB cases and overtreatment of individuals not having TB [19–21]. In 2011 WHO issued an unprecedented negative advisory on the use of such tests and the Indian government responded by banning the tests [22]. In reality, they remain widely available and continue to be aggressively marketed by companies. This landmark ban should be implemented region-wide and companies marketing unapproved tests for TB should be penalized to discourage the practice.

However, development and evaluation of novel diagnostic tests should not be discouraged. Commercial development of TB tests often suffers from lack of access to sputum from patients early in the development process. Prototype tests can consequently be manufactured for clinical evaluation which are unsuitable for use with real sputum or insufficiently sensitive to detect low numbers of bacilli after sputum processing. The high volume of potential TB patients screened in large clinics in South Asia provides an opportunity for the establishment of early diagnostic development centres, where prototype tests can undergo rapid early evaluation and feedback following standardized protocols. Such a diagnostic evaluation centre would reduce delays in obtaining ethical approval by having pre-approval to evaluate novel tests requiring only residual sputum samples and a rapid review process and standard commercial partnership agreement for each novel test requesting evaluation.

## Treatment

The patient burden in South Asia makes it ideally suited for the large scale clinical trials needed to evaluate TB regimens. Clear regulatory frameworks and procedures to obtain ethical consent can encourage clinical trials within countries while maintaining rigorous ethical standards [23]. The complexity of the clinical trials approval process in India has discouraged inclusion in multi-country trial networks. Recent changes have caused frustration but are an attempt to improve transparency [23]. By developing clear, transparent national guidelines to help academics and commercial organisations navigate the process and anticipate timeframes for approval, the number of clinical trials funded can be greatly increased.

The recent licensing of bedaquiline and delamanid is a major advance in our fight against drug resistant TB [24, 25]. However, licensing single drugs and allowing either unregulated use or reserving them for patients with the broadest resistance spectrums will foster the rapid emergence of resistance to our last line of defence. Balancing the needs of pharmaceutical companies to generate profits and the need for compassionate use of novel drugs in patients with no other options with the optimal public health strategies for protecting susceptibility to the drug is always a delicate balancing game. Governments and international agencies need to explore novel guarantees and incentives for pharmaceutical agencies, along the lines of the guaranteed ‘buy-down’ offered by UNITAID which facilitated the rapid roll-out of GeneXpert MTB/RIF. Attention is beginning to focus on the need for novel regimens rather than agents but may be too late as bedaquiline becomes more widely available. The Nix-TB trial has reported encouraging early results for a novel 3-drug regimen. Over the last 50 years we have actually preserved susceptibility to the limited number of potent antituberculous agents available remarkably well. This is largely due to the very early adoption of multi-drug therapy. By ‘locking’ new drugs into fixed dose combinations at the outset, we may more effectively preserve their utility. Structured rotation of first line regimens, as modelling studies have suggested as a strategy for malaria, may be another effective approach for the region, but will depend on the licensing of further novel agents [26].

In South Asia, pharmacies play a major role in the treatment of infectious diseases including TB with over- the- counter sale of many antibiotics with a very poor referral tradition even when the patient presents with classical symptoms of TB. In a recent study from India, only 13% of 599 pharmacies studied in Mumbai, Patna and Delhi refrained from selling antibiotics or steroids and properly referring a patient with two to 3 weeks of pulmonary TB symptoms [27]. Rules are in place but seldom followed. This behavior is characteristic of other countries such as Nepal and Bangladesh in this region. In another recent surprising finding from India, it has been shown that about two thirds of TB treatment may be handled by the private sector [28]. Although tuberculosis is a notifiable disease in many countries in South Asia including India, private practitioners are averse to notify [29] and there is no penalty involved. Worryingly, a study conducted in Dharavi, Asia’s most densely populated slum in the heart of Mumbai, India showed only 3% of the doctors practicing there were able to provide a correct prescription for a patient with MDR TB [28, 30].

Effective treatment depends on accurate diagnosis of drug resistance [31]. The GeneXpert scale-up has dramatically increased detection of MDR TB in the region. An 18 site Xpert implementation study demonstrated a 5-fold increase in resistant TB detection region [32]. The new, smaller more portable point of care Gene Xpert Omni scheduled for release in 2017, with a built in 4 h battery can potentially be used even in the far- flung regions of the Himalayas, Hindu Kush and Karakoram to improve confirmed diagnosis of TB and reduce unnecessary treatment. Until molecular drug susceptibility testing for drugs other than rifampicin improves, phenotypic drug susceptibility testing will remain necessary to appropriately treat MDR TB cases. Despite the population of 1.8 billion, the ratio of Drug Sensitivity Testing (DST) laboratories to population remains abysmally low at about 0.2 labs per 5 million population. Scale-up of quality-assured laboratory capacity is an essential component of any realistic attempt to combat drug-resistant TB in the region. It is hoped that improved methods for rapid whole-genome sequencing of tuberculosis deployable in resource limited settings may ‘leapfrog’ the need for substantial scale-up of phenotypic DST in the next decade (http://www.crypticproject.org/).

It is estimated that around 500 drug susceptible patients can be treated for the same cost as a single MDR case. Investing in strong MDR TB control is therefore highly cost effective if it successfully averts MDR transmission. In addition, the new shorter MDR TB treatment regimen from Bangladesh now recommended by the WHO should be implemented regionwide [33].

Strategies to improve TB patient outcomes must include evaluation of policies to facilitate adherence to treatment and improve outcomes. The application of mobile technologies to healthcare (M-health) has the potential for a transformational role in TB case management and reducing loss to follow up. Observation of treatment by video adherence (V-DOTS) can improve decentralization of treatment services and increase adherence. The role of M-health is under-explored in TB management [34].

TB is a disease entwined with poverty, and catastrophic household costs incurred by TB patients have been shown to be as significant a risk factor for default from treatment as MDR TB. Efforts are currently underway to map patient costs at national level led by WHO. The household burden and long-term repercussions can be alleviated by policies such as microcredit and cash transfer and optimal strategies need to be evaluated for scale-up in South Asia [35]. South Asia with rapid economic change has the potential to be at the forefront of interventions to eliminate catastrophic costs for TB [1].

## Prevention and infection control

The huge reservoir of latently infected individuals globally presents a substantial challenge to eradication efforts. Modelling of control strategies has consistently revealed that without tackling latent TB, eradication will not be feasible. For decades an unsubstantiated figure of ‘one third of the world’s population’ has been quoted as the number of people with latent TB, but a recently published study using annual risk of infection estimates derived from a number of sources produced a figure of 1.7 billion people, a quarter of the world population [36]. Scaling up isoniazid preventative therapy (IPT) in endemic countries has only received serious consideration in the last decade. For large scale IPT to be feasible, shorter regimens must be developed as well as regimens applicable in areas of high isoniazid resistance. The 12-dose, 3 month, rifapentine-isonaizid regimen is a step in the right direction but more must be done to evaluate strategies for IPT scale up beyond those currently being undertaken in people living with HIV. The interferon-gamma release assays (IGRA) tests for latent TB infection are impractical for large scale screening and do not predict those at greatest risk of developing active TB. Strategies for targeted prophylaxis need to be included in packaged interventions applicable to both urban and rural Asia [37]. Similarly, simple inexpensive infection control measures in healthcare settings including administrative changes, opening windows to improve ventilation or conducting question based screening of hospital attendees can protect healthcare workers and vulnerable patients from infection [38]. Such measures are not generally applied in South Asia and should be scaled up systematically through healthcare facilities in the region.

South Asia also has high vulnerability to natural disasters and political disruption. Contingency measures can go far in reducing the opportunistic spread of disease such as TB when disaster inevitably strikes. In the aftermath of the 2015 earthquake in Nepal, although funding was available a lack of capacity for delivery meant that suggested [39] simple healthcare measures were not delivered. Displaced people living in temporary, overcrowded shelters with no sanitation made millions of Nepalis vulnerable to infectious disease transmission, including TB and MDR TB. Damaged DOTS clinics and loss of drugs led to treatment interruptions for hundreds of TB patients.

## Research priorities

It is unlikely we will have an effective vaccine for TB within the next decade [40]. While we should not neglect vaccine research, we must make plans to advance towards TB eradication without a vaccine, rather than waiting for the ‘holy grails’ of a perfect vaccine or a substantially shortened regimen. South Asia is ideally and uniquely placed to contribute much needed strategic evidence towards the global eradication campaign.

Research priorities (Table 3) for the region must focus on packaged interventions both in large urban cities and remote rural populations. Alongside operational research to understand and optimize packaged interventions including patient incentives and social support, management and logistical strengthening and policy frameworks, early stage research should include robust diagnostic evaluation centres for early stage diagnostic development to complement the pipeline of demonstration/evaluation studies with FIND, an organization which works to improve the diagnosis of tuberculosis. Research capacity should be strengthened by enlarging the network of good clinical practice (GCP) compliant clinical trial sites to speed up novel regimen evaluations, improving laboratory capacity for bacterial culture and drug susceptibility testing, strengthening ‘omics capacity to understand susceptibility, transmission and drug resistance emergence. Networking research across the region to share and scale-up best practice, engaging governments and funders in the research agenda to ensure translation and political commitment to sustainable funding.

**Table 3:** Research priorities for TB in South Asia

Theme	Priority Research areas	AIMS
Diagnosis		
	Engaging the public sector to capture notifications and ensure treatment quality	Understanding components of successful Public Private Mix(PPM) models and resources needed for national scale-up
	Optimal algorithms and implementation behaviour	Improving application of optimal diagnostic algorithms.
	Facilitators and barriers to diagnostic access	Removing access barriers for TB diagnosis in underserved populations
	Childhood TB	Access to TB diagnosis for all children at risk
	Targeting Active Case Finding (ACF)	Understanding how to target ACF activities to maximise yield for minimal resources.
	Mobile health applications for TB	Minimising loss to follow up in diagnostic pathway and maximising outreach of novel diagnostics. Eg. Remote Chest Xray reading.
	Scale up of Drug Susceptibility Testing(DST) availability	Increasing access to full drug susceptibility testing through molecular scale-up and improving culture facilities in underserved populations.
	Integrating TB and diabetes	Understanding risk factors for TB among diabetic patients and optimal implementation of TB screening.
Treatment		
	Increasing Randomized Controlled Trial (RCT) capacity	Develop regional network and ability to systematically evaluate multiple regimens in adequately powered trials.
	Treatment of drug resistant TB, including INH mono-resistance, Multidrug Resistance(MDR) and Extensively Drug Resistance (XDR) TB.	Programme of trials to systematically answer locally relevant questions, including both alternative regimens and treatment delivery strategies
	Theoretical and practical studies to understand consequences of different approaches to novel drug roll-out	Optimising novel drug implementation in terms of negative outcomes averted and preventing emergence of resistance.
	Increasing drug resistance surveillance	Evaluating strategies applying molecular technology to increase monitoring of drug resistance prevalence and emergence
	Systematic evaluation of variation in pharmacokinetics and pharmacodynamics	Dose optimisation for existing drugs in adults and children to maximise efficacy, minimise adverse events and prevent resistance.
	Adverse drug events	Understanding susceptibility risk, improved detection and management of Adverse Events (AEs) and development of personalised regimens to avoid AEs.
	Retreatment regimen in non-MDR cases	Retreatment regimens that are economically feasible and effective allowing abolition of the currently failing ‘category 2’ retreatment regimen.
	Mobile health for Video Directly Observed Therapy (V-DOTS) and adherence	Alternative strategies for treatment monitoring to increase compliance while minimising costs and disruption to the patient.
	Optimal treatment of TB in diabetic patients.	Evidence base for optimal treatment and case management of TB in diabetic patients.
Prevention		
	Correlates of immunity	Understanding of components of a protective immune response for vaccine development.
	Standardising implementation of basic infection control in health care facilities regionwide	Development of evidence- based recommendations for minimal infection control standards in regional health care facilities.
	Targeted prophylactic therapy	Evidence base for risk:benefit of scale-up of prophylactic treatment in different population groups.
	Strengthening health systems resilience	Contingency measures for natural or political upheavals leading to person displacement or infrastructure loss
	Heterogeneity of risk in populations	Understanding molecular epidemiology of TB susceptibility and transmission in both dispersed rural and high density urban populations. Optimal targeting of TB control activities to appropriate risk groups.
	Risk of active TB in latently infected individuals	Biomarkers and social factors influencing risk for latently infected individuals developing active TB to optimise targeted prophylaxis.
	Host:pathogen interaction in susceptibility and transmission	Integrated analysis of host and pathogen genomes to understand complex interplay of host susceptibility and pathogen virulence.

## Conclusion

There is a clear opportunity as development in South Asia surges ahead to harness the power of the region’s skilled scientists and develop a regional research agenda for TB which encompasses the many facets of this complex problem. From bacterial biochemistry to policy implementation, South Asia has the patient numbers combined with a rich pool of innovative scientists which could enable it to seize the opportunity and lead global TB elimination by demonstrating feasibility in some of the world’s most densely populated cities and remotest reaches of the Himalayas. Clearly, political will and visionary leadership is of paramount importance, but TB will not be defeated by a single intervention and innovative measures will be essential. The time is ripe for researchers in the region to network together, develop a unified agenda and ensure successful research has a clear pathway to scale-up and impact towards TB eradication by 2050.
